# Cryopreservation of the Microalgae *Scenedesmus* sp.

**DOI:** 10.3390/cells12040562

**Published:** 2023-02-09

**Authors:** Martha Prieto-Guevara, Jany Alarcón-Furnieles, César Jiménez-Velásquez, Yamid Hernández-Julio, José Espinosa-Araujo, Víctor Atencio-García

**Affiliations:** 1 CINPIC, Fish Culture Research Institute, School of Veterinary Medicine and Zootechnics, Department of Aquaculture Sciences, University of Córdoba, Cra 6, 77-305, Montería 230002, Colombia; 2 School of Basic Sciences, Department of Biology, University of Córdoba, Cra 6, Montería 77-305, Colombia; 3 Faculty of Economics, Management and Accounting Sciences, Universidad del Sinú Elías Bechara Zainúm, Montería 230002, Colombia

**Keywords:** aquaculture, chlorophyceae, cryoprotectants

## Abstract

Each phytoplankton species presents a different behavior and tolerance to the cryopreservation process. Therefore, in a species-specific protocol, it is essential to ensure both growth and post-thawing cell viability. In this study, we explored the effect of cryopreservation of *Scenedesmus* sp. with two cryoprotectants, dimethyl sulfoxide (DMSO) and methanol (MET), at 5% and 10% inclusion for each. In the control treatment, the microalgae were not exposed to cryoprotective agents (Control). Three post-thawing cell viability criteria were used: no cell damage (NCD), cell damage (CD), and marked lesions (LM), and mitochondrial and cell membrane damage was evaluated by flow cytometry. The study was a 2 × 2 factorial design, with five replications by treatments, population growth, and cell damage evaluated from the fifth day after thawing. On the fifth day, the highest percentage of NCD was observed when the microalgae were cryopreserved with DMSO 5% (50%); Regarding the control group, it showed 0% NCD. Flow cytometry analysis reveals minor damage at the membrane and mitochondria (9–10.7%) when DMSO is used at both inclusion percentages (5–10%) after thawing. In the exponential phase, the highest growth rates, doubling time, and yield was observed in cryopreserved cells with MET 5%. The results suggest that DMSO 5% is an ideal treatment for cryopreserving microalgae *Scenedesmus* sp.

## 1. Introduction

Microalgae play an essential role in aquaculture as they form the basis of the food chain [1]. Microalgae have a significant nutritional and trophic value; approximately 90% of total aquaculture production is used as food at least at one stage of the development of cultivated organisms [2,3]. The most used are the species of green microalgae, widely worked as live food in the early stages of the larviculture of fish and freshwater crustaceans due to their nutritional value and easy production [4]. Among the microalgae used in aquaculture are Chlorophyta. The Chlorophyta comprise different genera, where *Scenedesmus* stands out thanks to its high nutritional value and growth rate, forming part of the *Scenedesmaceae* family. The genus has high levels of lysine, fatty acids, between 25 and 35% protein, and starch as a reserve nutrient [5]. *Scenedesmus* is planktonic, mainly in eutrophic freshwater ponds and lakes and rarely in brackish water. Currently, intensive production of microalgae is limited due to problems caused by loss or quality of the culture, and high operating costs which can reach half the cost of production [6,7,8,9]. In addition, traditional maintenance requires constant handling, aseptic conditions, time, and space [10]. However, despite the importance of developing and innovating in-process growth monitoring and control systems, most are suitable only for laboratory-scale use [11]. Therefore, it is necessary to have strains that meet the requirements of quality, health, and easy production [10]. In this way, biotechnological alternatives must be sought to keep the microalgae strains in optimal conditions and reduce the daily handling time, causing the least possible damage. In this sense, the cryopreservation of microalgae is emerging as an alternative for maintaining the unique characteristics of the strains over time.

Cryopreservation is a tool that is currently applied for the conservation of microalgae cultures [5,8,12]. This tool allows the storage and viable preservation of cells or tissues for long periods at cryogenic temperatures, usually between −80 °C and −196 °C. At these temperatures, a state is maintained in which any biological activity, including biochemical reactions that would result in a cell’s death, is effectively stopped. This is a fundamental process for conserving the cellular structure, as the inhibiting effects of low temperatures on the different physicochemical processes provide the necessary means to preserve the cell. However, some factors directly influence the process, such as concentration of cryoprotectants, and the break-even period, freezing rates, and thawing time, among others [5,8,12].

Cryoprotectants are water-soluble and low-toxicity substances which lower the eutectic point of a given solution. Biochemically, it is possible to distinguish three types of cryoprotectants: alcohols (methanol), sugars and dimethyl sulphoxide. Cryoprotectants can also be classified into penetrating and non-penetrating agents according to cell permeability [13,14]. Among the penetrating cryoprotectants, dimethyl sulfoxide (DMSO) is an aprotic, water-soluble, low molecular weight bipolar solvent. Its cryoprotective action is mainly attributed to its ability to prevent excessive accumulation of electrolytes and other substances during the freezing process. Likewise, it prevents the formation of ice crystals that break the structure of the membrane. Its low molecular weight allows rapid entry through the cell membrane, modulates the stability and phases of the phospholipid bilayer, and affects water solvation processes. For its part, methanol (MET) as a penetrating cryoprotectant is the simplest alcohol. The cryoprotective function of this substance is to protect the cell from damage that can be caused in the freeze–thaw process. This substance is widely used to lower the freezing point of the extracellular medium, minimizing the effects of ice crystals and favoring cell survival [14,15]. Additionally, it allows the immediate availability of strain for cultures. This process comprises three stages: balance, freezing, and thawing. To protect the cells throughout the process, cryoprotectants agents (CPAs) are added before freezing. During the equilibrium period, the microalgae–cryoprotectant suspension is maintained for a predefined time to allow exchange between intracellular solutes and the cryoprotective solution. Three essential variables relate to CPA use: concentration, temperature, and exposure time [8,12].

Despite all these advantages, cryopreservation techniques are unavailable or have not been well developed for all microalgae [5,8]. Therefore, the hypothesis of the present study was that the cryopreservation of the species *Scenedesmus* sp. is a biotechnological alternative for its maintenance. For the reasons above, the objective of the research was to explore and validate a cryopreservation protocol for the species to generate specific information that allows the development of cryopreservation techniques in microalgae of importance for aquaculture in Colombia and the neotropical region. The originality of this work lies in the development of an optimized protocol for the cryopreservation of microalgae. The importance of this work remains in the fact that the Microalgae Culture Collection of the University of Córdoba is a unique resource containing a diverse collection of microalgae isolated from the Sinu valley environment. Several cryopreservation techniques were applied to various strains to ensure maximum preservation of this resource, and the preservation effectiveness (cell viability) was determined.

## 2. Material and Methods

### 2.1. Localization and Obtaining the Strain and Handling

The study was carried out at the Fish Research Institute of the University of Córdoba (CINPIC), in the laboratories of Live Food, Cryopreservation of Fish Semen and the laboratory of Biology and Chemistry GRUBIODEQ, located in the city of Monteria, department of Córdoba, Colombia. The strain of *Scenedesmus* sp. was obtained from the Live Food laboratory of the CINPIC Fish Research Institute, University of Córdoba. The microalgae (1 mL) was cultured in test tubes with 9 mL of sterile water, nourished with F/2 medium from Guillard and Ryther [16,17] and maintained under direct light conditions with a fluorescent lamp (KL-1322 2X36W—2000 lux), 24 h a day at a constant temperature of 24 °C. The cultures in the exponential phase were centrifugated 3500 rpm for 10 min to obtain the concentrated microalgal biomass. Subsequently, the concentration was estimated with Neubauer plate counts (1/10 mm deep, Bright line-Boeco, Germany) under an optical microscope (Leica Microsystems, DM 500, Heerbrugg, Switzerland).

### 2.2. Cryoprotective Solutions

In the preparation of cryoprotective solutions, two cryoprotectants were used: dimethyl sulfoxide—DMSO (Sigma Chemical, San Louis, MO, USA) and methanol—MET (Sigma Chemical, USA) at two percentages and inclusions (5% and 10%) (Table 1). With the help of a micropipette (Transferpette^®^, CE704174, Baden-Württemberg, Wertheim, Germany) the volumes corresponding to each solution were taken. Thus, for the preparation of the cryoprotectants at a concentration of 5%, a total volume of 2000 μL (1900 μL of distilled water + 100 μL of cryoprotectant) was taken as a basis; in turn, for the preparation of the 10% cryoprotectants, a volume of 2000 μL (1800 μL of distilled water + 200 μL of cryoprotectant) was also taken as a basis. Subsequently, the mixtures to be frozen were prepared at room temperature (23 °C), and consisted of the addition of 20% concentrated microalgae + 80% cryoprotectant solution (this was based on the experiences of the research team in cryopreservation; the objective of this proportion is to provide protection and at the same time not generate toxicity on the cell [18]) for a total volume of 1 mL, arranged in Eppendorf of 2 mL for each treatment. Immediately, the biological material was packaged in straws of 0.25 mL. Four straws were made per treatment for a total of twenty straws. 

### 2.3. Freezing and Thawing

The cryopreservation of the microalgae *Scenedesmus* sp. was carried out in three stages: equilibrium period, freezing, and thawing. The equilibrium period, the mixing time between the microalgae and the cryoprotectant before freezing, was carried out for 30 min in complete darkness at room temperature (23 °C). Subsequently, the straws were frozen in a 4 L nitrogen steam thermos (dry shipper MVE, SC 4/2v, Philadelphia, Pennsylvania, USA) for 30 min. This time is considered an adequate equilibrium period for a cryopreservation process [8,19]. The cooling rate of nitrogen vapor was described by Cruz Casallas*,* et al. [20], thusly: from 28 to −20 °C, it drops to 27.3 °C/min; from −20 to −100 °C, it drops to 29.9 °C/min, and from −100 to −196 °C, the decrease is 5.5 °C/min until the cryopreservation temperature is reached (~−196 °C). The straws were then immersed in liquid nitrogen in a 34 L storage thermos for 48 h (MVE, XC 34/18, Minneapolis, Minnesota, USA). The groups of straws of the species were thawed in a serological water bath (Memmert, WNB 7–45, Baviera, Schwabach, Germany Germany) at 35 °C for 90 s. 

### 2.4. Analysis of Cell Viability after Thawing

Once the straws were thawed, the microalgae were inoculated in test tubes with 9 mL of sterile water nourished with Guillard and Ryther [16]’s F/2; they were then centrifuged at 3500 rpm for 10 min (Fisher, 225, Rochester, New York, NY, USA) to separate the microalgae from the cryoprotectant. Next, the concentrated microalgae biomass was inoculated into a new tube with enriched medium, from which five inocula of 1 mL were taken to perform five replications for each treatment, in which the viability of the cells and their population growth were recorded.

The viability of the cells was evaluated with the following criteria. The first was no cell damage (NCD), i.e., when the microalgae *Scenedesmus* sp. presented the central cells of the colonies with curved sides. At the same time, those located at the ends curved slightly to adopt a crescent shape. Usually, long appendages form a spine, a vibrant green color, an entire cytoplasm, a defined pyrenoid, a well-formed cell wall, chloroplasts, vacuoles, and visible starch granules. The next was cell damage (CD). This was considered to be when the microalgae *Scenedesmus* sp., despite preserving the number of cells and in some cases the mushrooms or spines, presented irregular shape; their cytoplasm was collected, they were without a defined pyrenoid. Their chloroplasts in some instances were not visible, and they had opaque coloration. The final criterion was marked lesions (ML). This was considered to be when *Scenedesmus* sp. Loses a number of cells in the row; isolated cells were observed, with an increase in size, deformity, and cytoplasm collected, and an accumulation of organelles in a sector of the cell, an invisible or undefined pyrenoid, rupture of the cell wall, and growth of the population of microalgae in culture. Cell counts were performed every three days in the Neubauer chamber to estimate the percentage of cells with different cell viability criteria. The rate of the viability of *Scenedesmus* sp. Cells was calculated immediately after thawing (day zero). On the fifth day, the recovery of the cells from damage caused by cryopreservation was evaluated. Subsequently, cell viability was assessed throughout culture (27 days).

The population growth curve throughout the culture was estimated by spectrophotometry after adjusting the absorbance with the help of the spectrophotometer, with a periodicity of three days in each of the 25 replicates until the microalgae reached its phase of decrease, in order to estimate the cell concentration for *Scenedesmus* sp. throughout 27 days of culture.

### 2.5. Flow Cytometry Damage Analysis

Flow cytometry (FC) is a procedure that allows multiparameter analysis of the suspended cell component in an individual, cell-by-cell manner, through its physicochemical characteristics, identifying the expression of cellular proteins and any cellular component or function that can be labeled with a fluorochrome. Flow cytometry allows you to measure the different parameters of a cell. These are divided into nuclear parameters, cytoplasmic parameters, surface parameters and extracellular parameters. Phytoplanktonic microalgae, both marine and freshwater, are mostly unicellular and rarely measure more than a few tens of micrometers in diameter, making them perfect candidates for easy analysis of their integrity of cytoplasmic and mitochondrial membranes using commercial flow cytometry equipment [21,22,23,24,25,26,27]. Part of the biological material of the microalgae *Scenedesmus* sp. thawed by each treatment (DMSO 5%, DMSO 10%, MET 5%, MET 10%, Control) was used in parallel to perform the respective flow cytometry studies and thus determine the integrity of the cytoplasmic and mitochondrial membrane. These biological samples were processed in the laboratories of the Cellular Immunology and Immunogenetics (GICG) group of the Cytometry Unit of the Medical Research Institute of the University of Antioquia, Medellín.

The straws cryopreserved with each treatment were thawed in a serological water bath (Memmert, WNB 7–45, Baviera, Schwabach, Germany) at a constant temperature of 35 °C for 90 s. Once thawed, they were deposited in 5 mL plastic tubes. Subsequently, 100 μL was taken with a micropipette (Transferpette^®^, CE704174, Baden-Württemberg, Wertheim, Germany) of the microalgae *Scenedesmus* sp. and deposited in another pipe, to which was added 400 μL of an enveloping solution (isotonic solution) plus 50 μL of a solution containing fluorochromes; 3,3′-dihexyloxocarbocyanine (DiOC_6_ (3)) (70 nm) + propidium iodide (PI) (Sigma, St. Louis, MI, USA) at a rate of 2 μg/mL with a micropipette (Transferpette^®^, CE704174, Baden-Württemberg, Wertheim, Germany. This was the case for each treatment. Subsequently, the corresponding tests were performed with a FACS CANTO II CYTOMETER (BD LSRFortessa™ San José, California, USA) by excitation of a 488 nm laser and fluorescence detection at 530/30 nm and 670 nm for DiOC_6_ (3) and PI, respectively. In the same way, fresh samples not cryopreserved were taken as controls, and the same management was given.

### 2.6. Population Parameters

Population growth was evaluated in the exponential phase, and the following parameters were determined:

Instantaneous growth rate (*k*)

k=(Ln (Nf)−Ln(N0) /t 

Doubling time (*td*)

td=Ln(2)/k

Yield (*r*)

r=(Nt1−Nt0)/t

where *Nf* represents the final cell number. *N*0 represents the initial cell number. *Nt*1 indicates the cell number at the end of the experiment. *Nt*0 indicates cell number at the beginning of the treatment. *t* means time.

### 2.7. Statistical Analysis

The cryopreservation of the microalgae species was carried out under a 2 × 2 factorial design. Five treatments were implemented with five (5) replicates per treatment (test tubes) for a total of twenty-five (25) experimental units. All recorded data regarding the variables studied were subjected to normality tests (Shapiro–Wilk tests) and homogeneity of variance (Levene tests). Data that met these assumptions had an ANOVA applied, and Tukey’s multiple range tests were applied when significant differences occurred. When the premises were not met, the data were subjected to Kruskal–Wallis nonparametric analysis. In all cases, 95% confidence (*p* < 0.05) was used as a statistical criterion to establish significant difference. Values were expressed as mean ± standard error. The statistical analysis was performed using IBM SPSS Statistics software, version 23.0.0.0, 2015.

## 3. Results

### 3.1. Post-Thawing Viability of Scenedesmus sp.

**Figure 1:**
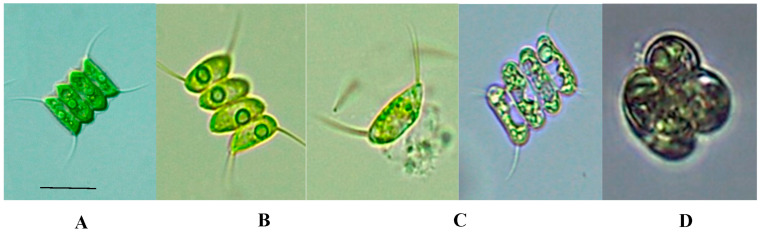
(**A**). (10 µm scale). Features of cells of *Scenedesmus* sp. post-thawing (**B**); no cell damage (NCD) (**C**); cell damage (CD) (**D**); marked lesions (ML).

**Figure 2:**
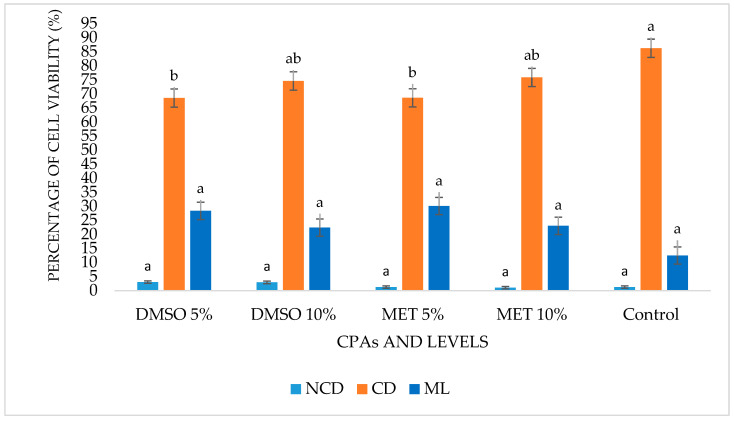
Percentage of cell viability of *Scenedesmus* sp. on day zero after thawing. No cell damage (NCD), with cell damage (CD) and marked lesions (ML) of the evaluated treatments. Different letters for the three criteria indicate significant differences (*p* < 0.05).

Figure 1 shows the viability criteria considered in this study. Figure 1A represents a fresh and non-cryopreserved microalga. Figure 1B illustrates no cell damage after the thawing process. Figure 1C characterizes cell damage, and Figure 1D denotes marked lesions of the cells. The results obtained with these criteria are the following: 

On day zero (0), the percentage of NCD cells ranged from 1% (MET 5%, MET 10%, and Control) to 3% (DMSO 5% and DMSO 10%) with no significant difference between these values (*p* > 0.05). The lowest percentage of cells with CD was recorded in DMSO 5% and MET 5% (69%) and the highest percentage in the Control treatment (86%) with a significant difference between these values (*p* ˂ 0.05). The percentage of cells with ML varied between 12% (Control) and 30% (MET 5%), with no significant difference between these values (*p* > 0.05) (Table 2 and Figure 2).

**Figure 3:**
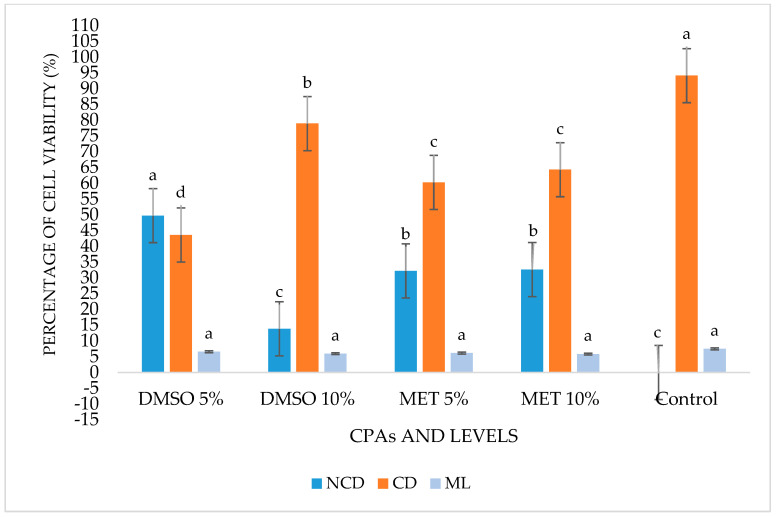
Percentage of cell viability of *Scenedesmus* sp. on the fifth day after thawing. No cell damage (NCD), with cell damage (CD) and marked lesions (ML) of the evaluated treatments. Different letters for the three criteria indicate significant differences (*p* < 0.05).

On day five, the highest percentage of NCD was observed using DMSO 5% (50%) and the lowest percentage in Control (0%), with a significant difference between these values (*p* ˂ 0.05). The lowest rate of cells with cell damage (CD) was recorded in DMSO at 5% (44%) and the highest percentage in Control (94%), with a significant difference between these values (*p* ˂ 0.05). The rate of marked lesions (ML) ranged from 3% (MET 5%) to 7% (DMSO 5%, DMSO 10%, MET 10%), with no significant difference between these values (*p* > 0.05) (Table 3 and Figure 3).

### 3.2. Damage to Mitochondria and Plasma Membrane in Scenedesmus sp. Post-Thawing by Flow Cytometry

On the first day after thawing, the highest percentage of viability and mitochondrial membrane potential was recorded in DMSO 5%, with 84.7% (Table 4), followed by DMSO 10%, with 81.8%; viability was observed at the mitochondrial level when using these treatments for the cryopreservation process. The treatment without cryoprotectant agents (Control), with 52.8%, presented the lowest percentage of viability in mitochondria.

On the other hand, the lowest percentage of damage at the cytoplasmic membrane level was presented by the treatment without cryoprotectant (Control), at 43.9%, followed by MET at 5%, with 17.5%, with little viability. The lowest percentage of damage was recorded by DMSO treatment, 10% with 9% (Table 4), followed by DMSO 5% with 10.7% (Table 4); high viability at the membrane level is observed when this cryoprotective agent is used in cryopreservation. The counts and characterizations by direct observation in Neubauer’s chamber showed that when using the DMSO 5% treatment, greater cell viability was obtained, which agrees with the results obtained by flow cytometry. 

### 3.3. Population Growth in Scenedesmus sp.

**Figure 4:**
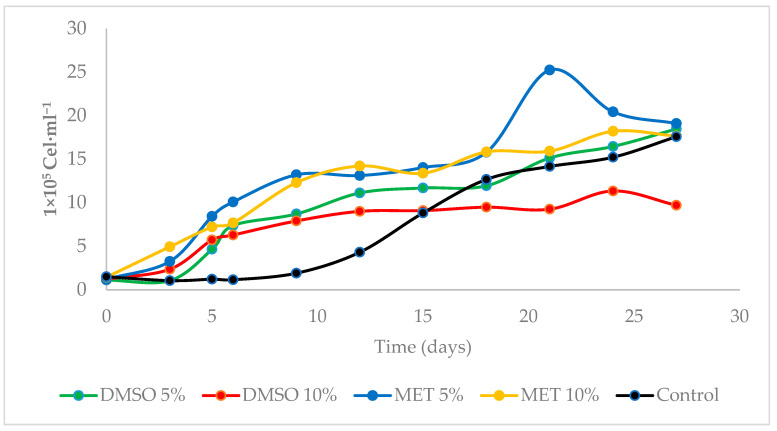
Adjusted population growth curve/real post-thawing of *Scenedesmus* sp., throughout the 27 days of cultivation.

The population growth of the microalgae *Scenedesmus* sp. throughout the twenty-seven (27) days of culture showed the different phases of growth in the five (5) treatments evaluated (Figure 4). At the beginning of cultivation, the first phase observed was the adjustment, registered until the third day for Control and DMSO 5% treatments, in which the microalgae adapted to the new growing conditions; meanwhile, for MET treatments 5%, DMSO 10% and MET 10%, an acceleration phase was observed in which cell division processes were initiated (Figure 4).

Subsequently, there was a phase of acceleration and exponential growth for all treatments from days 5 to 12 of culture (1st period), in which the population increased its number through cell division. Then, a phase of slowdown continued until the 15th day, and the population presented a reduction in its growth rate. Next, a second and final exponential phase was observed from days 18 to 24 of culture (2nd period) for all treatments, a period in which the populations registered their maximum density, as observed in Figure 4.

The experimental units showed a progressive decrease in the size of the population, characterizing the exponential descent phase, followed by a stationary phase; until day 27 of culture, cell density remained relatively constant in each treatment until it reached the death phase (Figure 4).

On the first day of culture, *Scenedesmus* sp. presented a similar density (*p* > 0.05); from the third day, the cells began to divide, and the population growth in the treatments showed a significant difference in density (*p* < 0.05) (Figure 4).

Thus, in the first exponential phase (days 1 to 12), the highest densities were recorded in MET 10% (1,422,666 ± 49,572 Cel.mL^−1^) and MET 5% (1,309,000 ± 123,762 Cel.mL^−1^), with a significant difference (*p* < 0.05) compared to DMSO 5% (1,112,666 ± 50,469 Cel.mL^−1^), DMSO 10% (850,666 ± 74,721 Cel.mL^−1^) and Control (542,000 ± 27,113 Cel.mL^−1^) (Figure 4).

Subsequently, in its second exponential phase (days 18 to 24), the population growth showed no significant difference between the treatments (*p* < 0.05), ranging from 1,886,000 ± 35,643 Cel.mL^−1^ (MET 5%) and 1,523,000 ± 459,464 Cel.mL^−1^ (Control). Finally, in the stationary phase (days 25 to 27), the highest density was observed in cryopreserved cells in Control (2,343,933.40 ± 432,089 Cel.mL^−1^), with a significant difference (*p* < 0.05) with DMSO 10% (1,339,866.80 ± 78,867 Cel.mL^−1^), which registered the lowest cell density (Figure 4).

**Figure 5:**
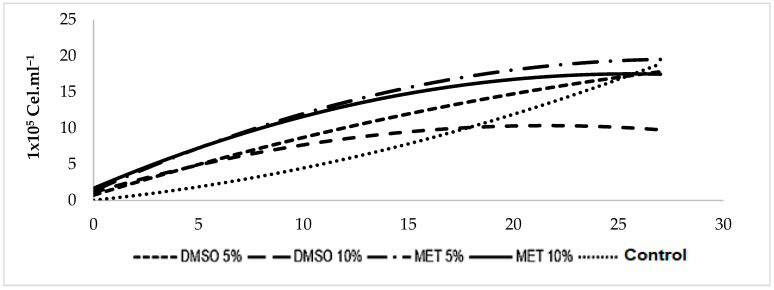
Adjustment models (trend lines) for post-thaw population growth curves of *Scenedesmus* sp. DMSO 5%. y = −976.4x*^2^* + 89,625x + 71,721, *r*^2^ = 0.9582; DMSO 10%. y = −1988.1x^2^ + 85,788x + 108,768, *r*^2^ *=* 0.944*;* MET 5%. y = −2327.6x^2^ + 130,268x + 129,845, *r*^2^ *=* 0.954*;* MET 10%. y = −2419.6x^2^ + 123,849x + 165,004, *r*^2^ *=* 0.9731*;* Control. y = 1490x^2^ + 29,658x + 1792.7, *r*^2 *=*^ 0.9479.

Figure 5 shows the projected density expected in the culture according to the model with the best fit: y = AX^2^ + BX + C + £. Where y is the value of the population density in time t and a, b and c are constants to estimate.

Regarding the population parameters of the microalgae *Scenedesmus* sp, the two periods corresponding to the exponential phase were evaluated throughout the population growth of each treatment. In the first period from days 1 to 12, cells cryopreserved in MET 5% registered the highest instantaneous growth rate k (0.20 ± 0.003) and the lowest doubling time td (3.55 ± 0.54), while MET 10% presented a higher yield r (106,444.44 ± 10,573); with significant differences (*p* < 0.05) in k and td with no CPA(Control) (Table 5). Thus, the Control treatment registered a lower k (0.10 ± 0.015), higher td (6.90 ± 1.20), and the lowest r (30,027.78 ± 8373), without significant difference (*p* > 0.05) in r with cryopreserved cells in DMSO 10%, but with the values obtained in DMSO 5%, MET 5% and MET 10% (*p* < 0.05) (Table 5).

The second period from days 18 to 24 of culture k, td, and r in the five treatments registered a progressive decrease compared to the first period; thus, microalgae cryopreserved with DMSO 10% registered a higher k (0.09 ± 0.059), lower td (−29.87 ± 88.893) and the highest r in MET 5% (77,222.22 ± 74,353). There was no significant difference between treatments (*p* > 0.05) (Table 5). On the contrary, the cryopreserved cells in Control presented the lowest k (0.008 ± 0.081), in DMSO 5%, the highest td (16.67 ± 9.157), and DMSO 10%, the lowest r (30,999.99 ± 48,784); there was no significant difference between treatments (*p* > 0.05) (Table 3).

## 4. Discussion

The microalgae *Scenedesmus* sp. registered a high percentage of DC (57%), with MET at 5%, and the highest population growth rate. With DMSO 5%, the highest percentage of SDC cells (50%) and adequate post-thawing population growth were recorded. In the cryopreservation of marine microalgae (*Nannochloropsis gaditana, Rhodomonas lens, Cylindrotheca closterium, Chaetoceros gracilis, Synechoccocus* sp. ans *Isochrysis aff. Galbana*); Paredes and Bellas [28] recommend using the lowest dose of cryoprotectant to reduce possible toxic effects. The authors found no significant differences between the two concentrations (DMSO 10% or 15%). Several authors suggest the lower concentration in the use of cryoprotectants to reduce toxic effects in the process of cryopreservation of microalgae [9,12,13,14,29].

Ali, Fucich, Shah, Hasan and Chen [9] reported that cryopreserved cells at low cryoprotectant concentrations obtained better viability rates than those at high concentrations. Based on the above, it can be affirmed that the type and concentration of cryoprotectants are species-specific. Therefore, the use of DMSO cryoprotectant in concentrations of 5% for the cryopreservation of the microalgae *Scenedesmus* sp. is suggested to obtain percentages of viability (SDC) greater than 50% on the fifth day of cultivation. However, this differs from what is reported by Jang [5], who asserts that 15% methanol is more suitable for the conservation of the genus *Scenedesmus.*

It is possible to infer that other critical factors in the cryopreservation process, such as equilibrium time and temperature, freezing and thawing curves, and tension in the centrifugation process, among others, could have affected the post-thawing viability in the cryoprotectants tested with the two concentrations (5% and 10%) [12,29,30]. In this sense, the levels of cell viability in the cryopreservation process vary depending on the protocol used and the species examined [8,9,13,14,29,31]. In this regard, when evaluating cryopreservation in four different species of the genus *Scenedesmus*, Jang [5] established that the species *Scenedesmus acutus* had the highest number of viable cells after growth, culture, and cryopreservation among the four strains; therefore, the cryopreservation protocol must be specific to each species and strain.

The temperature used in this research in the equilibrium period was 23 ± 1 °C. Temperatures between 10 and 25 °C during the break-even period are ideal for cryopreservation because they protect cells from CPA toxicity, as cryoprotectant is highly toxic at physiological temperatures. Other authors [8,31,32] indicate that temperatures between 18 and 25 °C facilitate the osmotic exchange of the DMSO solution and the cell during the equilibrium period, since the increase in temperature increases the molecular movement and kinetic energy of the solution, replacing the water molecules with the cryoprotectant substance. The temperature used in this research allows the cryopreserved microalgae *Scenedesmus* sp. the proper passage of cryoprotectant into the cell during the equilibrium period.

Toh, Liu, Tsai and Lin [31], and Kihika, Wood, Rhodes, Smith, Thompson, Challenger and Ryan [8] reported that the penetration rate of the cryoprotectant during the equilibrium period depends on the size and type of the cell as well as the differential permeability of cell membranes and the lipid content of the membrane. The break-even time in the research was 30 min. Therefore, the length of the effective equilibrium period varies from species to species. Another study [33] indicates that the duration of two equilibrium periods (15 and 45 min) with DMSO does not have a significant effect on the viability of *Chaetoceros calcitrans*; this could justify the low percentages of post-cryopreservation viability of the microalgae *Scenedesmus* sp. On the one hand, cells may not require extensive exposure times with cryoprotectants because they have a soft cell wall (*Ankistrodemus* sp.); on the other hand, for rigid cell wall cells such as *Scenedesmus* sp, the protocol allows for a timely balance between the cryoprotectant and the microalgae cells, with adequate recovery of these after their thawing.

The freezing protocol (nitrogen vapor for 30 min and subsequent storage in liquid nitrogen) has been used in the cryopreservation of various cells. Of this wide range of protocols, the two-step cryopreservation process has been reported to be among the most robust and reliable techniques, yielding high recovery levels; an added advantage of the two-step process is that it is easy and quick to perform compared to other methods, and does not require complex or expensive equipment [12,13,31]. Reference [12] suggested that a rapid cooling rate is more effective in freezing processes because the cells will be exposed for less time to possible changes in their structure. When the cells are subjected to slow freezing techniques, excessive dehydration is generated, causing a decrease in size and, subsequently, death. For his part, Jang [5] evaluated the three-step cryopreservation methodin one step: −40 °C (75 min), two steps: −80 °C (125 min), and three steps: −196 °C using liquid nitrogen; the author states that such a method is much more efficient in preserving cells.

The thawing technique used for this and other microalgae species (35 °C for 90 s) has been previously successfully evaluated in different cryopreservation protocols [18]. Other successful protocols for microalgae have also been recorded. For example, the straws thawed for 10 s in a 40 °C water bath [31], to a 37 °C water bath [14] and a 20 °C water bath until all visible ice melted [8]. When using fast freezing speeds, it is advisable to use a rapid thawing technique, avoiding cell recrystallization processes that can generate damage and consequently cell death.

Bui, Ross, Jakob and Hankamer [13] and [14] claim that the stress generated in the centrifugation process could sensitize the cell and decrease the likelihood of overcoming additional stress due to the cryopreservation process. Indeed, speed and time in the centrifugation process for removing cryoprotectants are paramount in the cryopreservation process. The speed and centrifugation time (3500 rpm × 10 min) used for the microalgae *Scenedesmus* sp. may have influenced the damage exposed throughout the culture period after thawing; since this genus has a soft cell wall [34], it is vulnerable to stress and generating genetic lesions in the microalgae. On the contrary, with those species with a more resistant cell wall, viable cells can be obtained more easily after thawing [28]. Therefore, based on the above, during the current investigation, the speed and time of centrifugation (3500 rpm × 10 min) used for the cells of *Scenedesmus* sp. are shown to be adequate by the good post-thawing recovery. For the reasons above, the current research suggests the optimization of cryopreservation protocols suitable for the maintenance of microalgae strains using new cryoprotectants in different concentrations and exposure periods, which is indispensable for the success of cryopreservation.

The determination of microalgal biomass by spectrophotometry can be beneficial to accompany and replace density counts that are hindered by the formation of chains, films, or aggregates. However, observing morphological indicators of the cellular condition of cultures under a microscope is indispensable. In this sense, direct cell observation through a microscope allowed us to establish that *Scenedesmus* sp. cryopreserved in MET 10% suffered lesions during the process that were then transmitted to the new cells. However, it presented the highest growth rate, doubling time, and yield of the microalgae with this treatment. In contrast, the growth of the strain of *Scenedesmus* sp. subjected to the process of cryopreservation in the present study shows a behavior and population growth similar to that of a traditional crop, which allows us to conclude that the conditions of the DMSO study in concentrations of 5–10% are adequate for cryopreserving *Scenedesmus* sp., allowing cell viability and good post-thawing population growth.

## Figures and Tables

**Table 1:** Cryoprotectors evaluated in the cryopreservation of the microalgae *Scenedesmus* sp. DMSO, dimethyl sulfoxide; MET, methanol; without cryoprotectant (control).

Treatments	Crioprotectants	Concentration (%)
DMSO5%	DMSO	5%
DMSO10%	DMSO	10%
MET5%	MET	5%
MET10%	MET	10%
Control		

**Table 2:** Results of the test between-subjects effect (ANOVA) for the factorial 2 × 2 design on day zero (0) after thawing.

Test between-Subject Effect						
Source	Dependent Variables	Type III Sum of Squares	df	Mean Square	F	Sig.
Corrected model	NCD	19.745 ^a^	4	4.936	0.762	0.562
	CD	1048.238 ^b^	4	262.059	3.257	0.033
	ML	950.708 ^c^	4	237.677	2.370	0.087
Intersection	NCD	87.626	1	87.626	13.521	0.001
	CD	140,242.769	1	140,242.769	1742.994	0.000
	ML	12,608.601	1	12,608.601	125.734	0.000
CPAs	NCD	16.907	2	8.453	1.304	0.293
	CD	4.018	2	2.009	0.025	0.975
	ML	8.307	2	4.154	0.041	0.960
Levels	NCD	0.000	0			
	CD	0.000	0			
	ML	0.000	0			
CPAs * levels	NCD	0.000	0			
	CD	0.000	0			
	ML	0.000	0			
Error	NCD	129.619	20	6.481		
	CD	1609.217	20	80.461		
	ML	2005.603	20	100.280		
Total	NCD	242.294	25			
	CD	142,397.352	25			
	ML	16,538.348	25			
Corrected total	NCD	149.364	24			
	CD	2657.455	24			
	ML	2956.310	24			

a. R squared = 0.132 (R squared fitted = −0.041). b. R squared = 0.394 (R squared fitted = 0.273). c. R squared = 0.322 (R squared fitted = 0.186). * means interaction between factors.

**Table 3:** Results of the test between-subjects effect (ANOVA) for the factorial 2 × 2 design on the fifth day (5) after thawing.

Test between-Subject Effect						
Source	Dependent Variables	Type III Sum of Squares	df	Mean Square	F	Sig.
Corrected model	NCD	7351.967 ^a^	4	1837.992	57.544	0.000
	CD	7353.377 ^b^	4	1838.344	61.408	0.000
	ML	47.011 ^c^	4	11.753	0.921	0.471
Intercept	NCD	14,313.477	1	14,313.477	448.125	0.000
	CD	120,268.865	1	120,268.865	4017.438	0.000
	ML	882.429	1	882.429	69.184	0.000
CPAs	NCD	1648.033	2	824.017	25.798	0.000
	CD	1940.781	2	970.391	32.415	0.000
	ML	25.481	2	12.741	0.999	0.386
Levels	NCD	0.000	0			
	CD	0.000	0			
	ML	0.000	0			
CPAs * levels	NCD	0.000	0			
	CD	0.000	0			
	ML	0.000	0			
Error	NCD	638.817	20	31.941		
	CD	598.734	20	29.937		
	ML	255.095	20	12.755		
Total	NCD	24,530.801	25			
	CD	124,511.533	25			
	ML	1201.386	25			
Corrected total	NCD	7990.784	24			
	CD	7952.111	24			
	ML	302.106	24			

a. R squared = 0.920 (R squared fitted = 0.904). b. R squared = 0.925 (R squared fitted = 0.910). c. R squared = 0.156 (R squared fitted = −0.013). * means interaction between factors.

**Table 4:** Percentage of cell viability in *Scenedesmus* sp. on the first day after thawing, measured by flow cytometry.

	Percentage of Viability in Mitochondria	Percentage of Damage in Membrane
DMSO 5%	84.7	10.7
DMSO 10%	81.8	9.09
MET 5%	74.3	17.5
MET 10%	78.8	12.8
CONTROL	52.8	43.9
FRESH	83.8	3.45

**Table 5:** Production parameters of the microalgae *Scenedesmus* sp. (± standard error) post-thawing: k, instantaneous growth rate; dt, doubling time; r, yield. Different letters between rows indicate a statistically significant difference (*p* < 0.05) between treatments.

Parameters	Treatments				
	DMSO5%	DMSO10%	MET5%	MET10%	Control
**1** **st period**					
k	0.19 ± 0.005 ^a^	0.17 ± 0.027 ^a^	0.20 ± 0.014 ^a^	0.19 ± 0.009 ^a^	0.10 ± 0.007 ^b^
dt	3.60 ± 0.103 ^b^	4.70 ± 0.865 ^b^	3.55 ± 0.240 ^b^	3.66 ± 0.160 ^b^	6.90 ± 0.535 ^a^
r	83,416.67 ± 3241 ^ab^	65,027.78 ± 14257 ^b^	99,166.67 ± 10646 ^a^	106,444.44 ± 4628 ^a^	30,027.78 ± 3745 ^c^
**2nd period**					
k	0.053 ± 0.012 ^a^	0. 09 ± 0.026 ^a^	0.047 ± 0.018 ^a^	0.027 ± 0.019 ^a^	0.008 ± 0.036 ^a^
dt	16.67 ± 4.095 ^a^	−29.87 ± 39.754 ^a^	3.30 ± 7.620 ^a^	−12.03 ± 18.707 ^a^	2.76 ± 4.848 ^a^
r	75,277.78 ± 17,996 ^a^	30,999.99 ± 21,817 ^a^	77,222.22 ± 33,251 ^a^	39,499.99 ± 29,826 ^a^	42,555.55 ± 45,480 ^a^

## Data Availability

All data provided in this manuscript were appropriately cited in the tables, figures, and reference section.
